# Long-term outcome in corneal endotheliitis with molecular detection of herpes simplex virus 1 and human herpes virus 6: a case report

**DOI:** 10.1186/s12886-022-02274-5

**Published:** 2022-02-02

**Authors:** Takashi Ono, Takuya Iwasaki, Yukiko Terada, Yosai Mori, Ryohei Nejima, Mineo Ozaki, Manabu Mochizuki, Kazunori Miyata

**Affiliations:** 1 Miyata Eye Hospital, 6-3, Kuraharacho, Miyakonojo, Miyazaki, 885-0051 Japan; 2 Department of Ophthalmology, Graduate School of Medicine, The University of Tokyo, Tokyo, Japan; 3 Department of Ophthalmology, Tokyo Metropolitan Geriatric Medical Center, Tokyo, Japan; 4 Ozaki Eye Hospital, Miyazaki, Japan

**Keywords:** Human herpes virus 6B, Herpes simplex virus 1, Corneal endotheliitis, Anterior uveitis, Case report

## Abstract

**Background:**

Human herpesvirus 6B (HHV-6B) is known to cause exanthema subitem and has been detected in various ocular diseases, including keratitis, uveitis, optic neuritis, and endophthalmitis; however, the long-term outcome after the reactivation of HHV-6B has not been well-addressed. Sugita et al. previously reported the concomitant presence of HHV-6B with herpes simplex virus-1 (HSV-1) in the aqueous fluid at the onset of corneal endotheliitis. We focused on the same patient with corneal endotheliitis, in whom both HSV-1 and HHV-6B sequences were observed, and reported the clinical course and long-term outcomes.

**Case presentation:**

A 64-year-old woman was referred to our center for visual disturbances in the left eye. Her best-corrected visual acuity in the left eye was 0.5 and the left intraocular pressure was elevated to 33 mmHg. Mid-sized keratic precipitates and 2+ cells were observed in the anterior chamber with corneal endothelial edema and reduction of the corneal endothelial cell density to 1828 cells/mm^2^. The patient was diagnosed with corneal endotheliitis with increased intraocular pressure. Polymerase chain reaction analysis revealed the concomitant presence of both HSV-1 and HHV-6B sequences in the left aqueous fluid. After treatment with oral valacyclovir and topical betamethasone, her intraocular inflammation gradually improved and has not recurred at 12 years after corneal endotheliitis onset although corneal opacity remained.

**Conclusions:**

Reactivation of HHV-6B infection might be associated with HSV-1 corneal endotheliitis; however, no serious late sequelae occurred after appropriate treatment for HSV-1 infection in this immunocompetent host.

## Background

Human herpesviruses have been known to become latent after primary infection and to reactivate. Human herpesvirus 6 (HHV-6), a T-lymphotropic β-herpesvirus, was first isolated from patients with a lymphoproliferative disorder [1]. HHV-6 is divided into two distinct viruses, HHV-6A and HHV-6B [2], which use different cellular receptors for virus entry (i.e., CD46 and CD134, respectively) [3, 4]. The clinical significance of HHV-6A infection has not been well demonstrated, although HHV-6B has been shown to be the major agent causing exanthema subitem (roseola infantum) as a primary infection [5], with > 90% of the general population developing a latent condition [6]. Reactivation of HHV-6B has been demonstrated to be related to encephalitis [7] and hypersensitivity syndrome in immunocompetent hosts [8, 9]. Furthermore, HHV-6B has been detected in various ocular diseases, including keratitis, uveitis, optic neuritis, and endophthalmitis; Sugita et al. performed multiplex polymerase chain reaction (PCR) analysis in 350 samples of uveitis or endophthalmitis and in 65 samples of corneal inflammatory diseases, and demonstrated that 2 and 1.5% of them were positive for HHV-6 DNA, respectively [10]. They concluded that HHV-6 infection or reactivation was implicated in ocular inflammatory diseases [10]. However, the long-term outcome after reactivation of HHV-6B in ocular inflammatory diseases has not been well-addressed to date. As corneal endotheliitis occasionally recur and may result in irreversible impairment of corneal endothelium, requiring corneal transplantation, it is essential for ophthalmologists to monitor patients for a long time. Therefore, in this work, we focused on one case of corneal endotheliitis, in which the concomitant presence of HHV-6B with herpes simplex virus-1 (HSV-1) was demonstrated [10]. Especially, we examined the clinical course and long-term outcome of a patient with corneal endotheliitis that was followed up for 12 years. Herein, in this report, we describe the 12-year outcome of the case described by Sugita et al.

## Case presentation

A 64-year-old woman was referred to Miyata Eye Hospital (Miyiazaki, Japan) for decreased visual acuity in her left eye. Intraocular inflammation with elevated intraocular pressure (IOP) had been observed in her left eye 10 months earlier at a local ophthalmology clinic. Despite treatment with topical steroids, it was difficult to decrease the IOP, and the patient was referred to us for management.

**Fig. 1:**
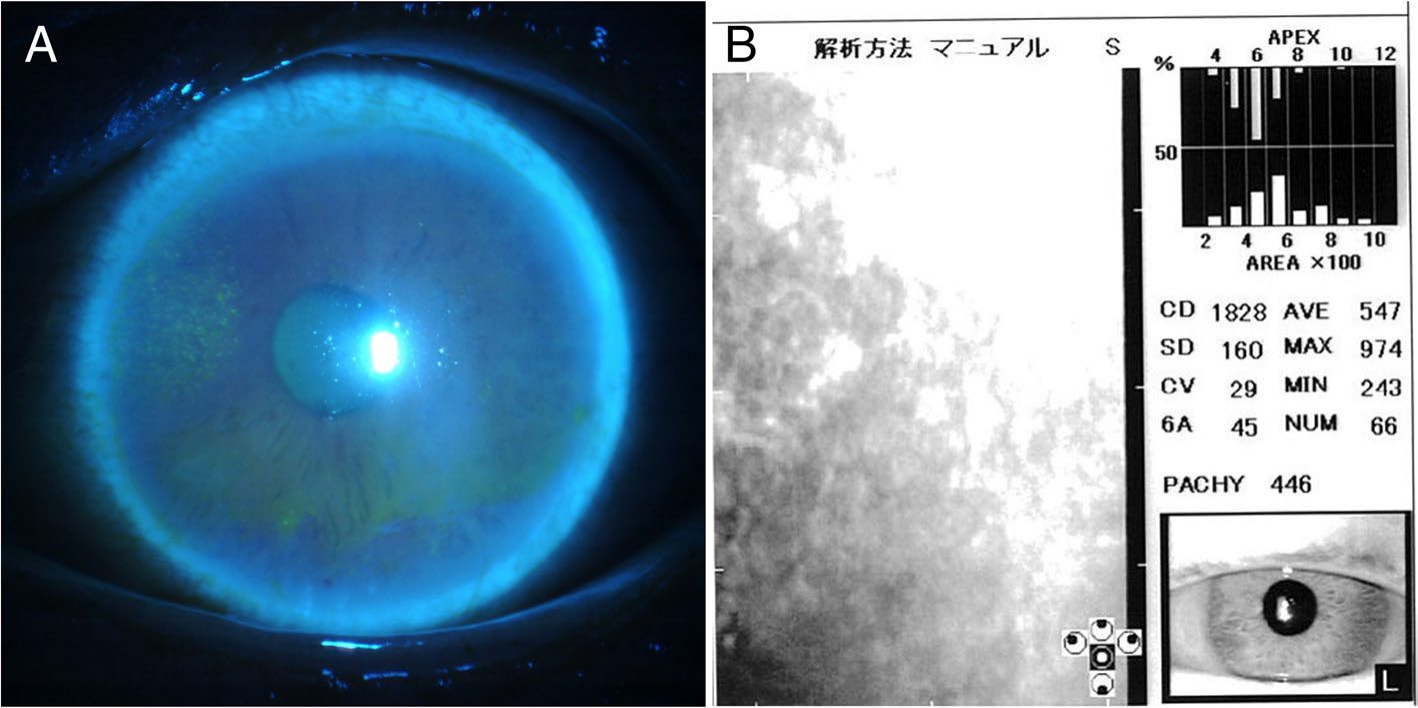
Slit-lamp examination photographs of a patient with corneal endotheliitis at initial presentation. **a** Anterior segment of the left eye with fluorescein staining showing corneal edema from the 9 to 12 o’clock position. **b** Corneal endothelial cell density and morphology of the patient’s eye at initial presentation

At initial presentation, slit-lamp examination showed hyperemia, mid-sized keratic precipitates (KP), and pigment KPs, 2+ cells in the anterior chamber of her left eye [10]. Focal corneal edema was also observed from the 9 to 12 o’clock position in the left eye with fluorescein staining (Fig. 1a); however, her right eye was normal. Additionally, mild cataracts were observed bilaterally. The best-corrected visual acuity (BCVA) was 0.5 and 0.4 in the left and right eyes, respectively; her IOP was elevated to 33 mmHg in the left eye and was 14 mmHg in the right eye. No corneal epithelial defects or ulcers were observed, and the corneal sensation was determined to be normal using a Cochet–Bonnet esthesiometer. Corneal endothelial cell density (ECD) decreased to 1828 cells/mm^2^, the coefficient of variation (CV) was 29%, and the appearance rate of hexagonal cells (6A) was 45% in the left eye (Fig. 1b) compared with 2058 cells/mm^2^ in the right eye. Anterior segment optical coherence tomography showed that central corneal thickness was 492 and 530 μm in the left and the right eye, respectively. Posterior segment inflammation was not observed and Goldmann perimetry revealed no visual-field defects.

The patient was subsequently diagnosed with corneal endotheliitis with increased IOP. Treatment was initiated with administration of topical betamethasone and levofloxacin four times daily, timolol 0.5% and travoprost 0.004% once daily, bunazosin 0.01% twice daily, and oral acetazolamide 750 mg/day. Since gonioscopic examination showed bilateral shallow angles, laser iridotomy (LI) was performed with an yttrium–aluminum garnet laser, which lowered the IOP to 21 mmHg.

Serological tests revealed the presence of HSV-1 IgG in enzyme-linked immunoassay and was negative for *Treponema pallidum* hemagglutination and for antibodies to human T-lymphotropic virus-1, varicella zoster virus, and cytomegalovirus. The blood sedimentation rate, C-reactive protein level, complete blood count, and electrolyte levels were within the normal ranges. Although she had a 3-year history of type 2 diabetes mellitus, it was well-controlled with a hemoglobin A1c level of 6.2%. She had no other systemic autoimmune disease and did not use oral steroids. The PCR analysis results of the anterior-chamber aqueous humor were previously reported by Sugita et al. [10]; briefly, HSV-1 and HHV-6B genome sequences were 2.8 × 10^5^ and 7.5 × 10^5^ copy/μL, respectively [10]. Accordingly, the patient was diagnosed with corneal endotheliitis and iritis with HSV-1 and HHV-6B. Oral valacyclovir was administered for 1 week at 3000 mg/day, followed by 1500 mg/day.

After 2 weeks, her IOP decreased to 11 mmHg, and topical bunazosin and oral acetazolamide were discontinued, although the 2+ cells in the anterior chamber persisted. As the cells decreased to 1+ after 1 month, oral valacyclovir administration was discontinued, and treatment with acyclovir 5% ocular ointment was initiated five times daily. At 2 months after initial presentation, the cells had completely disappeared, although the BCVA remained 0.5. Anti-glaucoma drugs were discontinued, with continuation of only betamethasone eyedrops and acyclovir ointment four times daily.

**Fig. 2:**
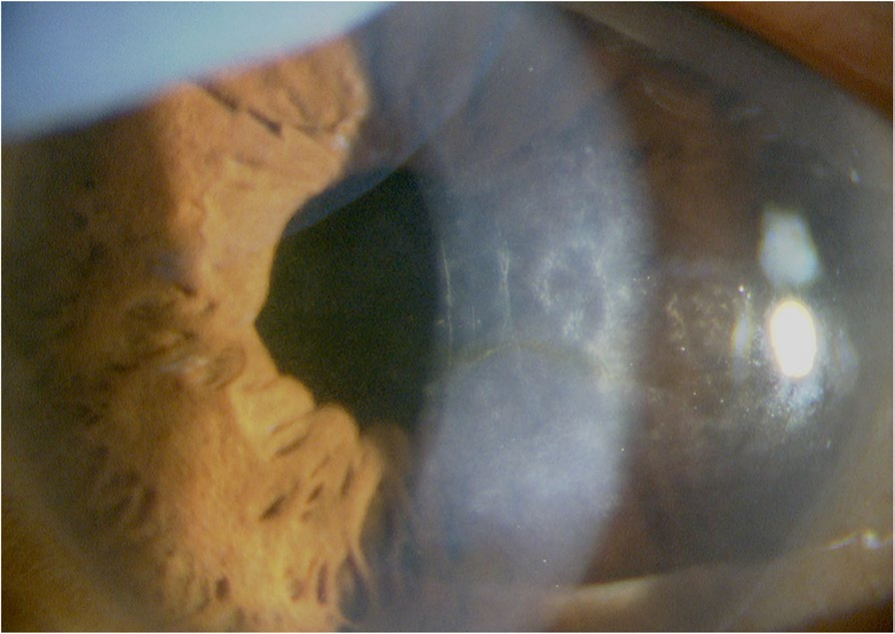
Slit-lamp examination photographs of the patient with corneal endotheliitis at final observation. Corneal opacity remained without recurrent intraocular inflammation at 12 years after corneal endotheliitis onset

Because of gradual progression of the cataract, surgery was performed in her left eye at 15 months after the initial presentation without exacerbation of intraocular inflammation; her BCVA improved from 0.4 to 0.8. Afterward, topical steroid administration, which was transiently increased to four times daily, was gradually tapered to betamethasone twice daily. At 6 years after the initial presentation, the ECD in her left eye decreased to 1698 cells/mm^2^, the CV was 60%, and 6A was 25%. Corneal endotheliitis did not recur with continued use of topical fluorometholone 0.1% and acyclovir ointment twice daily, although strong corneal opacity was observed. There was no vitreous opacity or inflammation in the fundus except for slight diabetic retinopathy of dot hemorrhage. At 12 years after the initial presentation, her BCVA was 0.5 owing to corneal opacity without recurrent intraocular inflammation or vitreous opacity (Fig. 2). The IOP was 17 mmHg with normal optic disc, and there was no retinal detachment or macular edema. Slight diabetic retinopathy was well controlled with HbA1c of 6.2%. Although counting the ECD was difficult in the opacified cornea, the central corneal thickness remained 410 μm.

## Discussion and conclusions

This case report describes the long-term outcome of a patient with corneal endotheliitis, in whom both HSV-1 and HHV-6B sequences were detected in the aqueous fluid and treated with valaciclovir. HHV-6 could be detected in various ocular inflammatory diseases [10]. However, to our knowledge, no such report describing the long-term outcome in HHV-6B-associated uveitis has been reported to date; therefore, our report would be beneficial for clinicians to describe the clinical manifestations, disease course, and treatment of the condition. This case showed that reactivation of HHV-6B in the aqueous fluid might be self-limiting and transient without serious consequences.

Our findings were consistent with those of corneal endotheliitis characterized by corneal edema and KPs, with mild anterior chamber reaction [11]. Corneal endotheliitis was originally considered to be an autoimmune disease; however, several viruses, such as HSV and cytomegalovirus, have been identified as its causative agents [12, 13]. In this case, the sequences of HSV-1 and HHV-6B genomes were detected in the aqueous fluid with cellular infiltration. HSV-1 was considered to be the principal cause for the condition because of the high copy numbers of viral sequences and good response to valaciclovir, which is effective in HSV-1 infection and ineffective in HHV-6B infection. Although HHV-6B might cause corneal endotheliitis, HHV-6B might be detected as a reactivated virus in infiltrating mononuclear cells in the aqueous fluid.

In the literature, HHV-6B has been detected in the ocular samples obtained from cases of corneal endotheliitis (*n* = 1; this case), bacterial keratitis (*n* = 1), bacterial endophthalmitis (*n* = 2), idiopathic uveitis (n = 1), Posner-Schlossman syndrome (*n* = 1) [10], and Behcet disease [14]. Considering its seropositivity, HHV-6B is considered to be reactivated in various ocular diseases. We could not clarify the pathogenicity of HHV-6B reactivation in this case. Further, no marked decrease in corneal endothelial density was observed during the 12-year follow-up period after the acute onset of the disease without HHV-6B-specific antivirals, such as ganciclovir. The long-term outcome of the patient in this case suggested that the self-limiting reactivation of HHV-6B was not responsible for the severe damage of the corneal endothelium.

HHV-6B is also known to be integrated into the human genome, and there may have been a possibility of detecting the integrated genome in this patient. Simple PCR analysis cannot differentiate between integrated and viral genomes, and we did not perform any analysis for HHV-6B integration in this patient; however, this should be confirmed before initiating HHV-6B-specific treatment. Previous reports have demonstrated that reactivation of HHV-6B may cause serious outcomes, such as bone marrow suppression, graft-versus-host disease, and encephalitis in hosts with immunosuppressed conditions, such as human immunodeficiency virus infection [15] and multiple myeloma [16], and in those undergoing chemotherapy [17]. Further, in case of detection of HHV-6B with PCR, the immune status of the patient should be assessed.

In this study, we described the long-term outcome of a patient with anterior uveitis and corneal endotheliitis, in whom the aqueous humor was positive for both HSV-1 and HHV-6. Secondary glaucoma with angle closure was appropriately treated with LI and anti-glaucoma medications. In this patient population, treatment with valacyclovir may be useful for preventing the recurrence of corneal endotheliitis, without performing any specific therapy for HHV-6B infection. It is therefore probable that HHV-6B has no clinical impact, merely being an innocent bystander.

## Data Availability

All data generated or analyzed during this study are included in this published article.

## Abbreviations

6A: Appearance rate of hexagonal cells
CV: Coefficient of variation
ECD: Endothelial cell density
HHV-6: human herpesvirus 6
HSV-1: herpes simplex virus-1
IOP: Intraocular pressure
KP: Keratic precipitates
LI: Laser iridotomy
PCR: Polymerase chain reaction
